# Increase in prevalence of *Streptococcus pneumoniae* serogroup 24 in children upon introducing 13-valent pneumococcal conjugate vaccine in Japan

**DOI:** 10.1099/acmi.0.000507.v3

**Published:** 2023-03-15

**Authors:** Misako Ohkusu, Kenichi Takeshita, Noriko Takeuchi, Naruhiko Ishiwada

**Affiliations:** ^1^​ Department of Infectious Diseases, Medical Mycology Research Center, Chiba University, Chiba, 260-8673, Japan

**Keywords:** *abpA*gene, sequence type, serogroup 24, *Streptococcus pneumoniae*

## Abstract

After introducing the 13-valent pneumococcal conjugate vaccine (PCV13) for children, a change in the prevalence of different *Streptococcus pneumoniae* serotypes that cause invasive pneumococcal diseases (IPDs) has been observed. The prevalence of vaccine serotypes has decreased and that of non-vaccine serotypes has increased. Currently, serogroup 24 has become one of the major non-vaccine serotypes causing IPDs in children in Japan. The aim of this study was to characterize clinical and genomic features of *S. pneumoniae* serogroup 24 strains isolated from sterile body sites in Japanese children. Serotyping, multi-locus sequence typing and genomic analysis of capsular polysaccharides of 61 strains of serogroup 24 were performed from 2015 to 2021. Among the 61 strains, 36, 23 and two belonged to serotypes 24F, 24B and 24C, respectively. The 24F sequence type (ST) 2572 and 24B ST 2572 were the major serotypes and sequence types observed from 2015 to 2019. By contrast, 24F ST 162 and 24B ST 2754 were the two major serotypes and sequence types observed after 2020. Two strains of serotype 24C were detected for the first time in Japan. Sequence analysis of the *abpA* gene, which plays a role in the synthesis of capsular polysaccharides in *S. pneumoniae *, was performed to distinguish different strains of serogroup 24. After the introduction of PCV13 in Japan, serogroup 24 has become one of the most prevalent non-vaccine serotypes causing IPDs in children. This serogroup has not been targeted in the next-generation pneumococcal conjugate vaccines. Therefore, monitoring of *S. pneumoniae* serogroup 24 that causes IPDs in children is essential.

## Data Summary

All new sequence data from this study have been submitted to the DDBJ/ EMBL/GenBank database under the following accession numbers: LC745951– LC745959, and LC746092–LC746152. The online version of this article contains Table S1 in supplementary material which shows the clinical information of serogroup 24 *S. pneumoniae* by sampling demographics.

## Introduction

*Streptococcus pneumoniae* is an alpha-haemolytic bacterium. In previous studies, microscopic examination of the pneumococcus revealed that it is a Gram-positive diplococcus, and a majority of *S. pneumoniae* are surrounded by a capsule. Currently, more than 100 capsular serotypes of *S. pneumoniae* are known [1]. *S. pneumoniae* is one of the leading causes of invasive bacterial diseases such as meningitis, bacteraemia and bacteraemic pneumonia [2, 3]. Pneumococcal conjugate vaccines [PCVs; heptavalent (PCV7), 10-valent (PCV10) and 13-valent (PCV13)] were developed to prevent invasive pneumococcal diseases (IPDs) in children. Recently, next-generation PCVs, namely 15-valent and 20-valent PCVs, were approved for prevention of IPDs in adults in the USA [4, 5]. These next-generation PCVs are expected to be introduced for children. After the introduction of PCVs for children, there has been a pronounced decrease in cases of IPDs caused by serotypes that are targeted by the PCVs [vaccine serotypes (VTs)] in many countries. However, the prevalence of non-vaccine serotypes (NVTs) of *S. pneumoniae* that cause IPDs has increased [6].

Serogroup 24 includes serotypes 24F, 24A, 24B and the newly identified 24C [7]. Serotype 24F is one of the most prevalent NVTs causing IPDs [8, 9]. The prevalence of serotype 24 differs among countries. A systematic review of serotype distribution of paediatric IPDs in the post-PCV era revealed that 24F is prevalent in Europe and the Western Pacific region, but not in North America [8]. Previously, it has been rarely isolated from children with otitis media and pneumonia in Japan, which suggests an increase in the invasiveness of this serotype [9]. A time series analysis conducted as part of a national survey in France demonstrated a sharp increase in the number of pneumococcal meningitis cases in children, which were primarily related to serotype 24F [10]. This phenomenon was observed between 2012 and 2014 after the introduction of PCV13. Although pneumococcal epidemiological change is influenced by not only vaccine pressure but also other internal and external factors, implementing a highly reactive surveillance system in each country is necessary to verify the local serotypic appropriateness of new-generation PCVs [10]. In Japan, PCV7 was introduced in February 2010, and administration of PCV13 as a routine vaccine began in November 2013. A survey of IPDs in children showed that the incidence of IPDs caused by serotype 24F increased considerably after the introduction of PCV13. In addition, we also found that the incidence of IPDs caused by serogroup 24 strains in children increased significantly after the introduction of PCV13 in Chiba Prefecture, Japan, while cases of IPD caused by serotype 24B strains have also increased since 2019 [11]. Therefore, a detailed analysis of serogroup 24 strains isolated from patients with IPD is required. Herein, we report the molecular analysis of *S. pneumoniae* serogroup 24 strains isolated from children after the introduction of PCV13 in Japan, mainly in Chiba Prefecture. In particular, we aimed to determine the genetic basis of structural differences in the capsules of pneumococci belonging to serotypes 24F, 24B and 24C.

## Methods

### Pneumococcal isolates

We collected *S. pneumoniae* strains from the cerebrospinal fluid or blood of paediatric patients with IPDs aged less than 15 years with IPDs. All these patients were admitted to various hospitals in Japan between January 2015 and December 2021. We collected samples from all children and adolescents with IPDs in Chiba Prefecture. Chiba is one of the 47 prefectures in Japan, located next to Tokyo. It has a population of 6.3 million, which accounts for approximately 5 % of the total population of Japan. Samples from nine other prefectures were obtained from clinicians on request, because of the lack of an active IPD surveillance system including bacterial analysis in the nine prefectures. Overall, we obtained 61 strains of *S. pneumoniae* serogroup 24 from sterile body sites of children with IPDs from various regions in Japan, covering 10 of the 47 prefectures. An IPD case was defined as the occurrence of *S. pneumoniae* in cerebrospinal fluid, blood or other normally sterile body sites.

### Preparation of chromosomal DNA

Bacterial strains were incubated overnight on blood agar medium supplemented with 5 % sheep blood at 35 °C in 5 % CO_2_. The colonies were then inoculated in 10 ml of Todd–Hewitt broth (THY) supplemented with 0.5 % yeast extract and grown to the mid-log phase. After incubation, the culture broth was centrifuged and the precipitates were extracted for chromosomal DNA isolation from each strain using MORA EXTRACT (Kyowa Hakko Industries). The concentration of chromosomal DNA was measured using a NanoDrop One (Thermo Fisher Scientific), and the concentration was adjusted to 100 ng µl^–1^. This genomic DNA was used for the PCR assay.

### PCR conditions

All PCR assays were performed with the same programme and reagent concentrations: 2× KOD One PCR Master Mix (TOYOBO), 0.3 μΜ of each primer and 0.5 µl DNA template mixed to a total volume of 25 µl. PCR was performed under the following conditions: initial denaturation at 98 °C for 1 min, followed by 30 cycles at 98 °C for 10 s, 55 °C for 5 s and 68 °C for 5–20 s, in a T-100 Thermal Cycler (Bio-Rad). Amplification was confirmed using electrophoresis of 3 µl of the PCR products in 1.5 % (w/v) agarose gels and visualized by staining with ethidium bromide.

### Sanger sequencing

The PCR products were cleaned using the FastGene Gel/PCR Extraction Kit (NIPPON Genetics) and used as samples for Sanger sequencing. Sanger sequencing of the PCR products purified on an ABI PRISM 3130xl Genetic Analyzer (Thermo Fisher Scientific) was performed using the BigDye Terminator v3.1 Cycle Sequencing Kit, at the Medical Mycology Research Center of Chiba University.

### Identification of *S. pneumoniae* serogroup 24 strains

All isolated strains were cultured on blood agar plates supplemented with 5 % sheep blood (Becton Dickinson) and incubated overnight at 37°C under 5 % CO_2_. Optochin susceptibility testing was performed using the disc diffusion method. The strains were streaked on sheep blood agar medium, and discs containing 5 µg optochin (Eiken Chemical) were placed on the blood agar medium and incubated overnight at 35 °C under 5 % CO_2_. According to the manufacturer’s instructions, the strains were identified as *S. pneumoniae* by confirming susceptibility with an inhibition zone of ≥14 mm. PCR assays targeting the *lytA* gene were performed to verify that the isolated strains belonged to *S. pneumoniae *. The isolates were serotyped by performing Quellung reactions using pneumococcal antisera (Statens Serum Institute).

### Multilocus sequence typing (MLST)

Sequence types (STs) were determined by comparing the sequences obtained from the analysed strains with those in the pneumococcal MLST database. Seven housekeeping gene sequences (*aroE*, *ddl*, *gdh*, *gki*, *recP*, *spi* and *xpt*) were amplified using sequences downloaded from the *S. pneumoniae* PubMLST database (RRID: SCR_012955 https://pubmlst.org/) [12].

### Molecular characterization of the gene encoding capsular polysaccharides (*cps*)

For the 61 strains, the *cps* gene of strain ST 2572 was analysed using PCR and DNA sequencing. For the PCRs, primers *FI3* (5′-TCTTAGTTCCATGGGATGCTTTCTGTGTG-3′) and *FI4* (5′-CGCTGAACTTTTGTAGTTGCTGTCTGGTCAAC-3′), and original primers were used [13, 14]. In *S. pneumoniae *, *cps* clusters (responsible for capsular biosynthesis) are generally located between *dexB* and *aliA* loci; thus, the primers *FI3* and *FI4* were designed using the sequences of these two flanking genes, to ensure that the intervening region containing the complete *cps* sequences could be amplified. PCRs were also performed with primers designed using the known sequences of genes in the *cps* locus of serogroup 24. The original primers were created using primer design software (Primer-blast) based on the sequences available on GenBank, accession numbers CR931688 (serotype 24F) and CR931687 (serotype 24B), which are known sequences at the *cps* locus of serogroup 24. The PCR products of the *cps* locus were sequenced using the Sanger method.

### Relationship between *abpA* genes of the *cps* loci and ST/serotype combinations

The *cps* sequences obtained in this experiment were aligned and compared with the reference *cps* loci *cps*24F (CR931688) and *cps*24B (CR931687) [15]. The *abpA* gene present in the *cps* locus was analysed in the 61 strains using PCR and DNA sequencing. Primers *8582F* (5′-CAGCTGGAAAGTTAATGGTTGGT-3′) and *9758R* (5′-ACCAATCAAACCAGAAGCTCCA-3′) were used in the PCR, following a published protocol [15]. Annotation of the determined gene sequences showed that the *cps* locus configuration of all serogroup 24 ST 2572 strains was the same as that of the gene used as a reference (CR931688). Sequence comparison revealed some scattered single nucleotide polymorphisms (SNPs) at the *cps* locus but no mutations common to strains 24F ST 2572 and 24B ST 2572 (except the *abpA* gene sequences). Therefore, we analysed the *abpA* gene sequences of isolates 24F, 24B and 24C that were isolated in this study. The PCR products were sequenced and the nucleotide sequence of the *abpA* gene was determined. The relationship between the *abpA* genes present in the *cps* locus and the ST/serotype combinations of the 61 strains was examined.

## Results

### Identification of *S. pneumoniae* serogroup 24 strains

The results of optochin susceptibility tests, PCR assays targeting the *lytA* gene and Quellung reactions using pneumococcal antisera confirmed that all 61 strains belonged to *S. pneumoniae* serogroup 24. Serogroup 24 was isolated in 31 of the 158 cases in Chiba Prefecture, and in 30 of the 112 cases in the other nine prefectures, which accounted for 19.6 and 26.8 % of all identified cases, respectively. A comparison of the sampling years, background of the patients and serotype/ST of the strains between Chiba and other prefectures is presented in Table S1 (available in the online version of this article). All 61 cases involved children aged 8 years or younger, with the majority of them <2 years of age.

### Serotypes and STs of the analysed strains

**Fig. 1.**
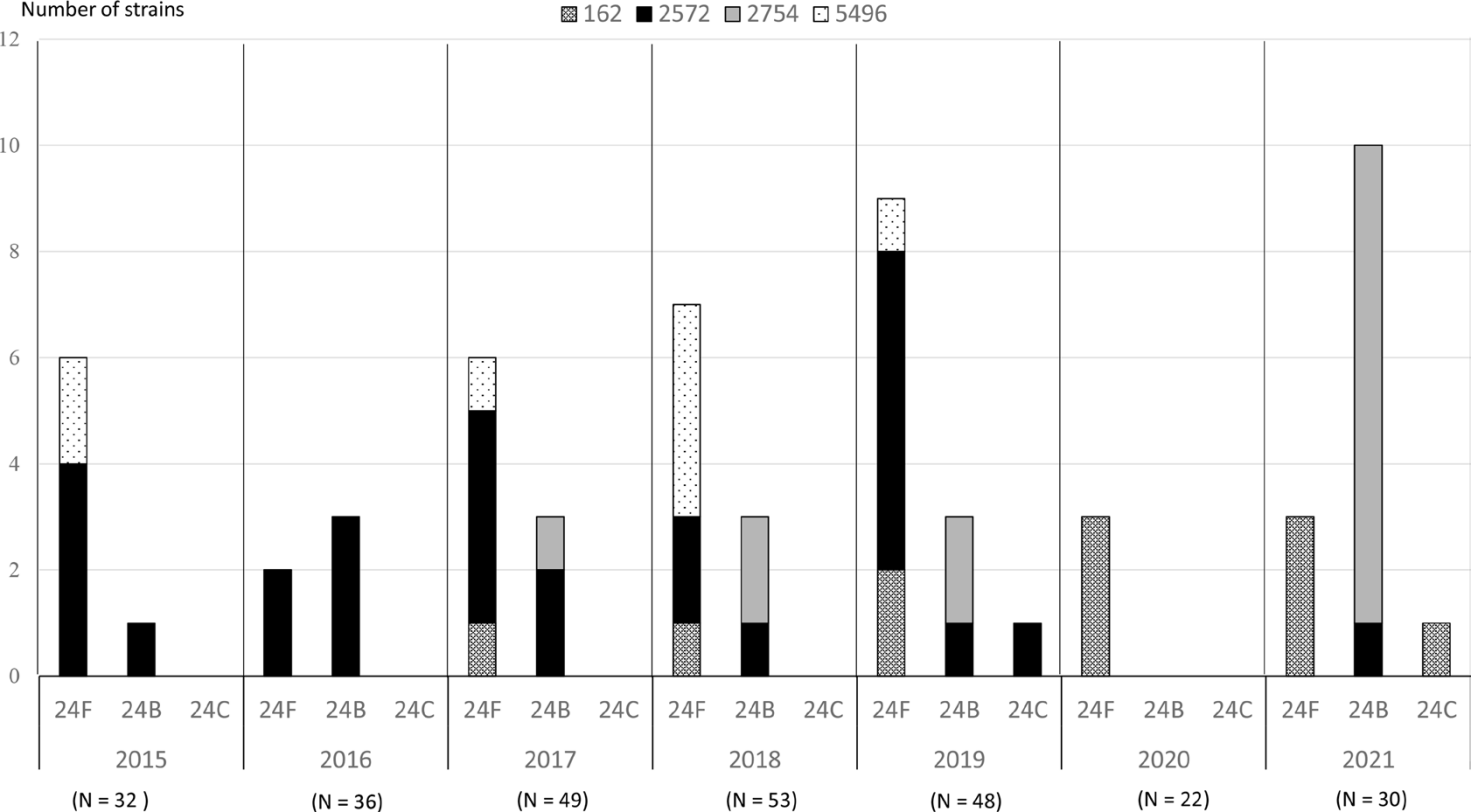
Annual change in the prevalence of different sequence types of *Streptococcus pneumoniae* serogroup 24 isolated from children with invasive pneumococcal disease (IPD). Among the 61 isolated strains, 36, 23 and two belonged to serotypes 24F, 24B and 24C, respectively. The number in parentheses is the total IPD cases each year. The *y*-axis gives the number of serogroup 24 strains.

Fig. 1 shows the yearly change in prevalence of different STs of serogroup 24 of *S. pneumoniae *, estimated using the samples isolated from children with IPDs. Among the 61 isolated strains, 36, 23 and two belonged to serotypes 24F, 24B and 24C, respectively. Serotype 24F was the major serotype in 2015 and from 2017 to 2020; however, in 2021, the major serotype was 24B. Three STs of serotype 24F were ST 162, ST 2572 and ST 5496. ST 162 was detected in all serotype 24F strains isolated in 2020 and 2021. ST 2572 and ST 2754 were initially observed in serotype 24B strains isolated in 2015 and 2017, respectively. In 2021, ST 2754 was detected in 90 % (9/10) of the 24B strains. Serotype 24C was isolated in 2019 and 2021. ST 162 and ST 2572 were found in the two serotype 24C strains. Serotype 24A was not detected during the study period. Overall, four STs were identified in serogroup 24 strains isolated during the study.

### Clinical information of the STs detected in *S. pneumoniae* serogroup 24 isolates

Table 1 shows the clinical information of the STs detected in the *S. pneumoniae* serogroup 24 strains isolated in this study. Most of these strains (48/61; 78.7%) were isolated from children <2 years of age. In terms of the clinical diagnoses, 40 strains were isolated from patients with bacteraemia and 11 from patients with bacteraemic pneumonia. Five strains were isolated from patients with meningitis: two 24F ST 162 strains, and one strain each of 24C ST 2572, 24F ST 2572 and 24F ST 5496. Serotype 24F ST 162 was isolated from patients of diverse age groups and various clinical diagnoses.

**Table 1.** Clinical information regarding the sequence types detected in *S. pneumoniae* serogroup 24

Sequence type	Serotype	No. of strains	Age distribution (no. of strains)	Clinical diagnoses (no. of strains)
162	24F	10	<2Y (5) 2–5Y (4) >5Y (1)	Bacteraemia (5) Meningitis (2) Pneumonia+pericarditis (1) Arthritis (1) Cellulitis (1)
162	24C	1	2–5Y (1)	Bacteraemia (1)
2572	24F	18	<2Y (15) 2–5Y (3)	Bacteraemia (14) Pneumonia (2) Meningitis (1) Cellulitis (1)
2572	24B	9	<2Y (8) 2–5Y (1)	Bacteraemia (6) Pneumonia (3)
2572	24C	1	<2Y (1)	Meningitis (1)
2754	24B	14	<2Y (11) 2–5Y (3)	Bacteraemia (8) Pneumonia (6)
5496	24F	8	<2Y (5) 2–5Y (2) >5Y (1)	Bacteraemia (6) Meningitis (1) Infective endocarditis (1)

Y, years.

### Relationship between *abpA* genes of the *cps* loci and ST/serotype combinations

The blast search results revealed that the *cps* loci (except the *abpA* gene sequences) of all serogroup 24 strains shared 100 % identity. The *cps* loci (except the *abpA* gene sequences) of all strains of 24F ST 2572 and 24B ST 2572 were identical.

**Fig. 2.**
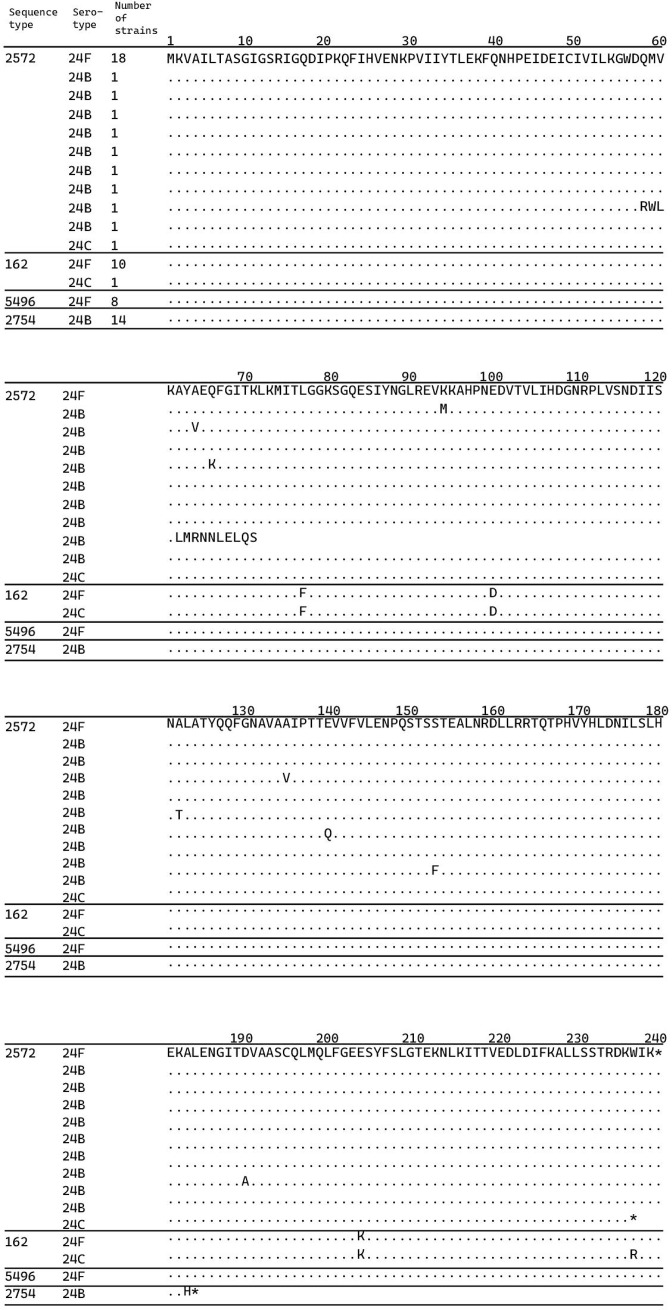
Sequence type (ST), serotype and strain number of *Streptococcus pneumoniae* and multiple alignments of amino acids of the protein encoded by *abpA*. ST 2572 was detected in serotypes 24F, 24B and 24C; ST 162 in serotypes 24F and 24C; ST 5496 in all serotype 24F strains; and ST 2754 in all serotype 24B strains. Isolated strains with more than one strain listed had the same amino acid. Amino acid alignment was performed using the amino acid sequence of ST 2572, with serotype 24F as a reference. Amino acids identical to those of the reference sequence are indicated with dots, and sequences differing from the reference are indicated with uppercase letters. Numbers above the alignment indicate the position of the amino acid. Asterisks indicate stop codons.

Therefore, we analysed the *abpA* gene sequences in strains 24F, 24B and 24C that were isolated in this study. The *abpA* genes of the 24F ST 2572 strain had identical nucleotide sequences. Fig. 2 shows the relationship between *abpA* sequences and ST/serotype combinations; the analysis was performed using the *abpA* gene sequence of 24F ST 2572 strain as the reference sequence. The *abpA* gene sequence was used to distinguish among 24F, 24B and 24C strains. The C-terminal sequence of the *abpA* gene was different between the two 24C strains. All 24B ST 2754 strains had an identical single nucleotide deletion in the *abpA* gene sequence, causing a frameshift. On the other hand, each of the nine 24B ST 2572 strains had a different point mutation in the *abpA* gene, causing an amino acid substitution. We also found that ST162 had differences shared across all ST162 isolates and these were not present in other STs. We observed diversity within ST 2572 serotype 24B, while the recently emerged serotype 24C was identified across at least two STs.

## Discussion

After the introduction of PCVs, the incidence of IPDs caused by VTs has decreased dramatically in many countries. On the other hand, the incidence of IPDs caused by NVTs has increased [6, 16]. Among all the NVTs, serogroup 24 was one of the most prevalent serotypes in Japan [11]. Surprisingly, serogroup 24 is a major serogroup causing IPDs in children, but it has been rarely isolated from adults with IPDs in Japan [17, 18]. This has also been reported in France [19]. However, in Denmark, the IPD caused by serotype 24F has increased not only in children but also adults [20]. Serogroup 24 is categorized into four serotypes, namely 24F, 24A, 24B and 24C. Among them, serotype 24C has been recently identified [7]. In this study, we analysed the relationships between serotypes and ST combinations of several serogroup 24 strains and their clinical manifestations.

In this study, serotypes 24F, 24B and 24C were isolated from children with IPDs. Serotype 24F was the major serotype causing IPDs from 2017 to 2020. The results obtained from the MLST analysis demonstrated the prevalence of specific STs in all the isolates. During the study period, in the serotype 24F strains, the major ST was initially ST 2572, which changed to ST 5496 and then to ST 162. Serotype 24F ST 2572 was mainly isolated from children aged <2 years of age with bacteraemia. A survey of paediatric IPDs in Japan revealed that the prevalence of serotype 24F ST 2572 isolates increased significantly between 2012 and 2014 [9]. According to data available on the PubMLST database, serotypes 24F ST 2572 and 24F ST 5496 (a single-locus variant of ST 2572) were recorded only in Japan. By contrast, serotype 24F ST 162 was isolated from patients of various age groups with various types of IPDs, including meningitis. In a recent study, serotype 24F ST 162 was reported to be the major ST in serotype 24 strains isolated from patients with IPDs in Denmark [20]. Furthermore, Cao *et al*. [21] reported the incidence of severe pneumococcal meningitis caused by serotype 24F ST 162 in Hong Kong. A unique characteristic of *S. pneumoniae* strains belonging to serotype 24F ST 162 is that they are CO_2_-dependent and cotrimoxazole-resistant, a characteristic which is also observed in *S. pneumoniae* strains belonging to serotype 9V ST 162 [22]. In our study, all 61 strains were susceptible to penicillin G, whereas 24F ST 162 strains were cotrimoxazole-resistant. In the future, it will be essential to closely monitor the trends associated with serotype 24F ST 162.

Interestingly, the major serotype changed from 24F to 24B during the study period, especially serotype 24B ST 2754, the prevalence of which increased rapidly in 2021. Most of the serotype 24B ST 2754 strains were isolated from children <2 years of age with bacteraemia or bacteremic pneumonia. Serotype 24B ST 2754 has not been reported from other countries. These findings suggested that this phenomenon was caused by the clonal spread of serotype 24B ST 2754 in Japan, similar to the one observed in the case of serotype 12F ST 4846 [23]. However, to clarify the origin of 24B ST2754, further studies are needed.

This is the first report on the isolation of serotype 24C from patients with IPD in Japan. In this study, only two serotype 24C strains were identified, the first of which was isolated in 2019. These two 24C strains had different STs. Serotype 24C ST 2572 was isolated from a patient with meningitis. Until now, serotype 24C has been isolated from patients in Germany and England [7]. Our findings suggest that serotype 24C has spread to various regions of the world.

Previously, variations in the sequences of the *abpA*, *abpB* and *wcxG* genes were the reason for the differences observed in the subtypes of serogroup 24 [15]. However, a recent study has revealed that these sequence variations are not serotype-specific. Ganaie *et al*. [7] analysed the sequence of an approximately 2.6 kb region of the *cps* loci (including the *wcxG*, *abpA* and *abpB* genes) of several strains representing the four related serotypes 24F, 24A, 24B and 24C. Gene sequences of *wcxG* and *abpB* in these four serotypes were similar to the published sequences, and no serotype-specific differences were observed. By contrast, the *abpA* sequences were not identical among all four serotypes [7]. Therefore, we analysed the *abpA* gene sequences in serogroup 24 strains isolated in this study. The *abpA* gene, formerly known as *abp1*, is 720 bp long and encodes a transferase enzyme (240 aa) that is involved in the biosynthesis of CDP-arabinitol [24, 25]. In our study, all 18 strains of serotype 24F ST 2572 had the same amino acid substitutions in the sequence of the protein encoded by the *abpA* gene. By contrast, there is considerable diversity within serotype 24B ST 2572. Analysis of the *abpA* gene sequence helped distinguish between 24F and 24B types of the ST 2572 strain. The C-terminus of the *abpA* gene in both 24C strains was different from that of the 24F ST 2572 strain. This change in the C-terminus of the *abpA* gene was observed in the 24C ST 162 strain, but not in the 24F ST 162 strain. Comparison with the reference *abpA* sequence of the 24F ST 2572 strains revealed point mutations in the *abpA* sequences of all 24B ST 2754 strains, whereas no mutations were observed in the *abpA* sequences of any of the 24F ST 5496 (single-locus variant of ST 2572) strains.

The genetic variability of serotypes 24F, 24B and 24C is an interesting phenomenon. One possibility is that serotype 24F strains initially colonized the nasopharynx, and then human-to-human transmission occurred. During this invasion process of different body sites, the 24F serotype mutated to either the 24B or 24C strain [26]. In other words, when the 24F strain invaded deeper tissues, it might have encountered the host immune response that targeted arabinitol. Thus, the 24F serotype switched to the 24B or the 24C serotype to avoid an immune response to the host [27]. Another possibility is that immunologically related pneumococcal capsular serotypes were concomitantly present in the same patient. The simultaneous recovery of two serotypes of serogroup 24 from exudates of the middle ear and blood culture samples has been reported [28]. However, these proposed mechanisms underlying the variations have not been fully demonstrated. Considering the above hypothesis, a thorough investigation of serogroup 24 strains isolated from sterile body sites and the nasopharynx is necessary. Further studies are needed to clarify the precise mechanisms involved.

This study has several limitations. First, a nationwide survey was not conducted and the regional differences in Japan were not considered in this analysis. Although sampling from Chiba Prefecture was population-based, requested samples from the other nine prefectures may have been biased. Second, the sampling period included the time after the introduction of PCV13. We investigated MLST of 11 serogroup 24 strains isolated during 2010–2014. Among them, eight strains belonged to serotype 24F ST 5496, and one strain each belonged to serotype 24F ST 2572, serotype 24F ST 9609 (one locus mismatch of ST 2572) and serotype 24B ST 2572. However, we could not perform a precise analysis of the strains before the introduction of PCV13. Finally, we could not conduct whole genome sequencing of the strains nor analyse the factors responsible for the invasiveness of the isolated strains.

In conclusion, we analysed *S. pneumoniae* serogroup 24 strains isolated from children with IPDs in Japan. This study was conducted after the introduction of PCV13 in Japan. Serotype 24F ST 2572 was the major serotype causing IPDs from 2017 to 2019. By contrast, 24F ST 162 and 24B ST 2754 were the two major serotypes observed after 2020. Two strains of serotype 24C were collected during the study period. Analysis of the *abpA* genes present in *cps* loci helped in distinguishing between different strains of serogroup 24. After the introduction of PCV13 in Japan, serogroup 24 is one of the most prevalent serogroups causing IPDs in children. This serogroup is not targeted by the next generation of PCVs, namely 15-valent and 20-valent PCVs. We need to closely monitor the clinical features associated with serogroup 24 in the future.

## Supplementary Data

Supplementary material 1
