# Supplementary material for: Increase in prevalence of Streptococcus pneumoniae serogroup 24 in children upon introducing 13-valent pneumococcal conjugate vaccine in Japan
Source: Access Microbiol. 2023 Mar 15;5(3):acmi000507.v3. doi: 10.1099/acmi.0.000507.v3 (PMC10118250; doi:10.1099/acmi.0.000507.v3)
Supplement: Supplementary material 1 [file acmi-5-507.v3-s001.pdf]

Supplemental Table 1 Clinical information of serogroup 24 *S. pneumoniae* by sampling demographics

Sampling site (Number of the strains)	Chiba prefecture (31)	Other prefectures (30)
Name of the prefecture	Chiba (31)	Tokyo (14) Aichi (7)<br>Shizuoka (3) Saitama (1)<br>Nagasaki (1) Fukui (1)<br>Kyoto (1) Oita (1) Nagano (1)
Sampling Year	2015 (5) 2016 (6)<br>2017 (5) 2018 (4)<br>2019 (4) 2020 (1)<br>2021(6)	2015 (1) 2016 (0)<br>2017 (4) 2018 (6)<br>2019 (9) 2020 (2)<br>2021 (8)
Age distribution	0Y (5) 1Y (20) 2Y (2)<br>3Y (1) 4Y (3)	0Y (4) 1Y (16) 2Y (6)<br>3Y (1) 5Y (1)<br>7Y (1) 8Y (1)
Clinical diagnoses	Bacteremia (17)<br>Pneumonia (9)<br>Cellulitis (2)<br>Meningitis (1)<br>Infective endocarditis (1)<br>Arthritis (1)	Bacteremia (23)<br>Meningitis (4)<br>Pneumonia (2)<br>Pneumonia+pericarditis (1)
Serotype and sequence type	24F 2572 (12)<br>24F 5496 (4)<br>24F 162 (2)<br>24B 2572 (6)<br>24B 2754 (7)	24F 2572 (6)<br>24F 5496 (4)<br>24F 162 (8)<br>24B 2572 (3)<br>24B 2754 (7)<br>24C 162 (1)<br>24C 2572 (1)

Abbreviation: Y; years
